# Linking Learning Environment and Critical Thinking through Emotional Intelligence: A Cross-Sectional Study of Health Sciences Students

**DOI:** 10.3390/healthcare11060826

**Published:** 2023-03-11

**Authors:** Antonios Christodoulakis, George Kritsotakis, Panagiotis Gkorezis, Panayota Sourtzi, Ioanna Tsiligianni

**Affiliations:** 1 Department of Social Medicine, School of Medicine, University of Crete, 71003 Heraklion, Greece; 2 Department of Business Administration & Tourism, Hellenic Mediterranean University, 71309 Heraklion, Greece; 3 Department of Economics, Aristotle University of Thessaloniki, 54453 Thessaloniki, Greece; 4 Department of Nursing, National and Kapodistrian University of Athens, 11527 Athens, Greece

**Keywords:** critical thinking disposition, emotional intelligence, learning environment, healthcare students

## Abstract

Background: Health sciences educators should increase the critical thinking of their students as this may improve the quality of care. However, this is rarely considered as a critical point in teaching, despite studies identifying factors such as the learning environment and emotional intelligence as increasing critical thinking at an undergraduate level. Thus, there is a need to better explore these factors and investigate interrelations and ways of improving critical thinking, especially in the critical field of healthcare students (nursing and medicine). Objectives: The present study aimed to examine the potential relationships between critical thinking with emotional intelligence and the learning environment. Method: This was a descriptive cross-sectional study with 208 first year health sciences university students of two nursing departments and one medicine department from three universities in Greece. The Critical Thinking Disposition Scale, Dundee Ready Education Environment Measure, and Trait Emotional Intelligence-Short Form questionnaires were used to assess critical thinking, the education environment, and emotional intelligence, respectively. Results: The results demonstrate that critical thinking was positively related to emotional intelligence (β = 0.82, *p* < 0.001), but not to the learning environment (β = 1.06, *p* = 0.30). However, a structural equation modeling analysis supported the indirect relationship between the learning environment and critical thinking through emotional intelligence (M = 1.10, CI = 0.13–2.17, *p* < 0.05). Conclusions: Emotional intelligence may be the underlying mechanism for achieving critical thinking if it is well applied and cultivated in a learning environment. Therefore, universities could modify their curricula and place emotional intelligence at the epicenter of teaching.

## 1. Introduction

Healthcare professionals attempt to deliver high-quality care in demanding and changing environments [1]. This attempt is demanding as there is an increase in ageing, with complicated and new healthcare needs [2]. To manage these complications, healthcare professionals need a set of skills, such as critical thinking, patience, teamwork, empathy, and communication [3]. These skills should be cultivated at the earliest possible time, usually during undergraduate studies, and enable healthcare professionals to deliver high quality care [4]. 

Critical thinking is considered as the foundation for the development of clinical skills [5]. It can be defined as: “a deliberate, self-regulating thinking that leads to interpretation, analysis, evaluation, and conclusions” [6,7]. By definition, critical thinking can accelerate the diagnostic process, improve decision making, augment medical nursing procedures, and expediate problem solving in everyday clinical practice [8]. Due to the numerous advantages of critical thinking, healthcare professionals are expected to cultivate it from their university years and utilize it later during clinical practice [9]. However, studies indicate a low [10,11] to moderate [12] increase in critical thinking in health sciences students at the undergraduate and post-graduate levels. 

Several factors can influence critical thinking. Ideally, educators should be able to identify and improve these factors to better cultivate the critical thinking of their students [13,14,15,16,17,18,19,20]. These factors can be classified into two categories [2,20,21,22]: (a) modifiable and (b) unmodifiable. Some examples of modifiable factors are the learning environment, self–confidence, emotional intelligence, and area of expertise [16,17,19]. The most common unmodifiable factors are age, prior individual experiences, gender, and ethnicity [13,14,20]. Thus, medical educators should target and increase these modifiable factors during training [23,24,25]. Of these modifiable factors, improving the learning environment [17,19,20,26] and increasing emotional intelligence [27,28,29,30] can also improve clinical skills, even at the undergraduate level. 

The learning environment can be conceptualized as “the social interactions, organizational culture and structures, and physical and virtual spaces that surround and shape the learners’ experiences, perceptions, and learning” [31]. The learning environment promotes critical thinking by incorporating specific educational methods [26,32], such as problem-based learning [1,33], reflective writing [34], concept mapping [35], and case studies [36]. Furthermore, the learning environment can also increase students’ emotional intelligence by incorporating other methods, such as flipped classroom teaching and learning and e-learning platforms, with online forums and activities [37]. Emotional intelligence is another modifiable factor of critical thinking [21]. It is defined as the ability to recognize, understand, and manage your emotions [38]. Undoubtedly, high levels of emotional intelligence are related to better management outcomes [29,30] and a higher quality of care [27,28]. 

**Figure 1:**
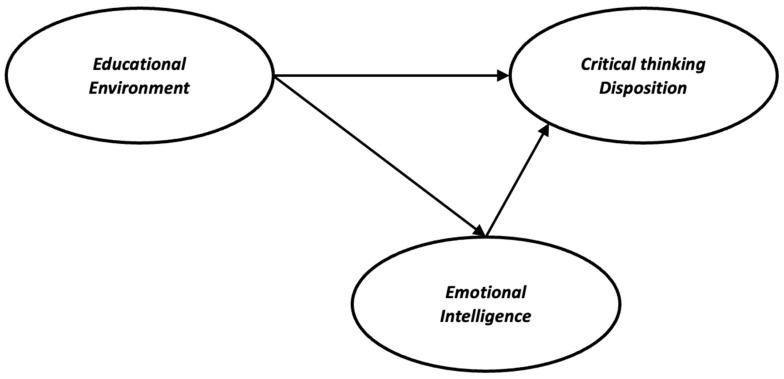
The hypothesized model of the study.

In summary, a modified learning environment may increase critical thinking [32] and emotional intelligence [39] at the individual level. Studies suggest a positive relationship between critical thinking and either the learning environment [17,19,20] or emotional intelligence [27,28,29,30]. Moreover, previous studies have examined the role of the learning environment in facilitating critical thinking. However, to date, limited empirical research has focused on the mechanisms by which this effect occurs. Thus, the present study attempts to contribute to this gap by examining the indirect relationship between the learning environment and critical thinking through emotional intelligence. In doing so, we also provide new insights into the outcomes of emotional intelligence and the antecedents of critical thinking. Therefore, we examined three main hypotheses. First, the learning environment is positively related to critical thinking disposition in pre-graduate healthcare students. Second, critical thinking disposition is positively related to emotional intelligence among healthcare students. Finally, we hypothesized that emotional intelligence would mediate the relationship between the learning environment and critical thinking disposition in healthcare students (Figure 1).

## 2. Materials and Methods

### 2.1. Design and Sample

This study had a descriptive cross-sectional design. All 346 first-year university students of two nursing departments and one medicine department in three Greek universities were asked to participate in this study. In this population, a confidence level of 95% and a margin of error of 5% would be achieved with a sample size of 183 healthcare students. After obtaining permission from each class’s professor, the students were asked to participate in the study. Participation was voluntary, and every student signed a consent form. The inclusion criteria were (a) being a first-year student and (b) being willing to participate in the study. There were no exclusion criteria. Among the 346 first year students, a convenience sample of 208 students who agreed to participate (60% response rate) was included in the present study. 

### 2.2. Instruments

The students provided their key sociodemographic characteristics (age, gender, and university) in a self-reported questionnaire and completed the following three scales for each of the study variables (critical thinking, emotional intelligence, and learning environment). 

#### 2.2.1. Critical thinking Disposition Scale (CTDS)

We measured critical thinking by estimating the disposition for it. Critical thinking disposition is considered to be a valid way for measuring critical thinking, since having an ability implies having the disposition to utilize it [40,41,42,43,44]. Furthermore, Sosu (2013) elaborates that having critical thinking disposition is a “way that an individual reasons, argues, and makes decisions” [40]. It should be noted that CTDS was selected because it is a brief scale that would be able to be used in everyday academia in Greece since, to the best of our knowledge, there are not any translated and validated scales that measure the critical thinking disposition in the Greek language.

The English version of CTDS comprises 11 questions of two dispositional domains/factors assessed on a 5-point Likert scale in which a higher score is indicative of higher critical thinking, with a range of 11–55 [40]. The first factor, ‘*Critical Openness*’, depicts the extent to which a person is open to new ideas, evaluates them, and modifies existing ideas when enough evidence is procured. The second factor, ‘*Reflective Skepticism*’, illustrates the ability of a person to learn from past experiences and examine the validity of evidence [41]. CTDS has been used in healthcare students in China, Spain, and the US, showing adequate psychometric properties [45,46,47]. 

The Critical Thinking Disposition Scale (CTDS) was translated and adapted in Greek for this study. The process of adjusting CTDS to Greek was completed in three steps [48]. Firstly, three bilingual (English andreek) experts translated the English version into Greek. After that, a fourth translator addressed any differences in the preliminary translations, resulting in a final translation. The final Greek version of the CTDS was then back-translated into English by two bilingual professors who had not seen the original form of the English scale. After that, any dissimilarities between the translated versions were assessed and appropriate adjustments were made. The final Greek version was evaluated for its validity and reliability in a sample of 30 students. The data collection for the test–retest process was performed in a separate group of final year nursing students that were not otherwise involved in this study, with an intermediate completion time of 13 days. Between phases 1 and 2, Pearson’s r was estimated for the total score (r = 0.726, *p* < 0.001) and for the first (r = 0.560, *p* = 0.004) and second factors (r = 0.820, *p* < 0.001) (‘*poor-to-good reliability*’). Exploratory factor analysis (EFA) with principal component analysis (PCA) and varimax rotation with Kaiser normalization were also performed to explore the original scale’s structure in Greek and its equivalence to the initial scale. The Kaiser–Meyer–Olkin (ΚΜO) measure of sampling adequacy was estimated at 0.77 (middling fit) and the Bartlet’s test of sphericity determined that the dataset was appropriate for factor analysis (χ^2^ = 506.7, d.f. = 55, *p*-value < 0.001). Initially, four components or factors with eigenvalues ≥ 1.00 and 63.0% interpretation of the total variance were detected. However, according to Sosu’s definition [41], two components or factors were selected that interpret a total of 44% of the total variance, with high eigenvalues but also higher loadings in determining the items for each factor (a minimum loading criterion of 0.40 was adopted in order for scale items to be maintained in each factor). Additionally, internal consistency was examined using Cronbach’s alpha.

#### 2.2.2. Dundee Ready Education Environment Measure (DREEM) 

Dundee Ready Education Environment Measure (DREEM) [49] assesses the quality of the learning environment and has been translated and validated into Greek [50]. The DREEM consists of 41 positive statements (each rated from 0 to 4) and 9 negative statements (rated from 4 to 0). DREEM comprises 5 subscales, namely (1) students’ perceptions of teaching (12 items), (2) students’ perceptions of teachers (11 items), (3) students’ academic self-perceptions (8 items), (4) students’ perceptions of atmosphere (12 items), and (5) students’ social self-perception (7 items). It creates an overall score and five sub-scales on student’ perceptions of learning and their teachers, their academic perceptions, perceptions of the institution’s atmosphere, and their social perceptions. On all subscales, the higher scores indicate a good learning environment: the higher they are, the better [51]. It should be noted that DREEM was chosen since it measures the most easily modifiable aspects of learning environment and can be used to examine potential relationships with other questionnaires [52]. It should be noted that the 9 negative statements of the DREEM were reversely entered into the data set that was used for the analysis.

#### 2.2.3. Trait Emotional Intelligence Questionnaire Short Form (TEIQue-SF) 

Trait Emotional Intelligence Questionnaire-Short Form (TEIQue-SF, [53] is the short form of TEIQue [54], which assesses 15 trait emotional intelligence facets. Furthermore, it has 4 subscales: (1) well-being, (2) self-control, (3) emotionality, and (4) sociability. Participants respond on a 7-point Likert scale (1 = “strongly disagree”—7 = “strongly agree”). TEIQue-SF has been translated and validated in Greek and has been found to be satisfactory for measuring emotional intelligence [55,56]. Because this is the short form, the subscales tend to have lower internal consistency than the full form. However, the short version has much lower completion time, thus making it ideal for use in studies with multiple questionnaires such as this one.

### 2.3. Data Collection 

Most of the samples (*n* = 160) were collected during classes. However, 48 students completed the questionnaires on a different day, in an online platform specifically designed for our study. The platform was provided and is maintained by one of the participating universities. Students who consented to participate received the questionnaires during classes or a link in their academic mails. Data were collected between December 2019 to February 2020. The survey took approximately 30 min to complete.

### 2.4. Ethical Considerations

In accordance with ethical principles, all data have been pseudoanonymized and securely stored. Students, before giving their written informed consent, were also reassured that their participation in the study—or lack thereof—would not influence their academic scores, that their data would be anonymous, and that they could withdraw from the study at any time. To utilize each questionnaire, written permission was received via separate emails from each author. 

### 2.5. Data Analysis

Data were analyzed using the SPSS software (version 26.0, SPSS Inc., Chicago, IL, USA). A normality test was performed on the scales according to Blom’s method (Q-Q plot). The frequencies of the descriptive characteristics of 208 students and their answers in the scales’ questions were estimated, as well as the scores’ descriptive measurements and reliability coefficients (Cronbach’s alpha). Acceptable Cronbach’s alpha scores were from 0.70 to 0.95 [57]. The Pearson correlation coefficient was used to measure the relationship between scales and subscales. In the case of CTDS, it was also used as an index of convergent validity. Based on the categories of low/moderate and high disposition of CTDS, Student t test was used to compare the levels of DREEM and TEIQue-SF scales. Finally, hierarchical modeling with multiple linear regressions was performed for CTDS and each DREEM and TEIQue-SF scale/subscale, controlling for basic personal characteristics of the 208 first year healthcare university students. We also used a three-step approach to test our third hypothesis [58]. This approach introduces three conditions, and, when they are met, mediation occurs. The conditions were (a) the independent variable relates to the dependent variable, (b) the independent variable relates to the mediating variable, and (c) the mediating variable relates to the dependent variable, and the relationship of the independent variable with the dependent variable is significantly lower in magnitude (or insignificant) in the third equation than in the second. Additionally, we conducted bootstrap analysis (5000 bootstrap samples, 95% confidence intervals) in SPSS using a macro named “PROCESS” [59]. The advantage of this analysis is that we do not assume normality in sampling distribution.

## 3. Results

The study sample comprised 208 university students, of which, 25.5% were male and 74.5% female (Table 1). The majority were between the age of 18–20 (86.1%) and studied nursing (68.7% vs. 31.3% medicine) (Table 1). 

Most of the students had a moderate to high critical thinking disposition (mean score 44.5 ± 4.9 SD) (Table 2). Furthermore, the Reflective Skepticism sub-scale had a lower mean score (mean score 15.2 ± 2.2 SD) than the Critical Openness subscale (mean score 29.3 ± 3.5 SD) (Table 2).

Most of the students had a more positive than negative overall perception of the learning environment (mean score 124.4 ± 20.4 SD) (Table 2). Moreover, students scored higher on the “Learning” (mean score 29.0) and “Atmosphere” (mean score 29.8) subscales, and lower on the “Academic” (mean score 21.3) and “Social” (mean score 17.8) subscales of the questionnaire (Table 2). 

Students had a moderate to high emotional intelligence. More specifically, the TEIQue-SF mean score was 4.92 (± 0.66 SD) and students scored higher on the “Well-being” (mean score 5.25) and “Emotionality” (mean score 5.03) subscale and lower on the “Self-control” (mean score 4.4) and “Sociability” (mean score 4.66) subscales (Table 2). 

Reliability was assessed based on Cronbach’s alpha (0.570–0.933), which was acceptable (overall Cronbach’s α: CTDS = 0.783, DREEM = 0.933, and TEIQue-SF = 0.823), in all scales/subscales (Table 2). 

The learning environment had a positive effect on the emotional intelligence of health sciences students (β = 0.82, *p* < 0.001). However, the regression analysis showed that the learning environment did not relate to critical thinking (β = 1.06, n.s.) (Table 3). 

**Figure 2:**
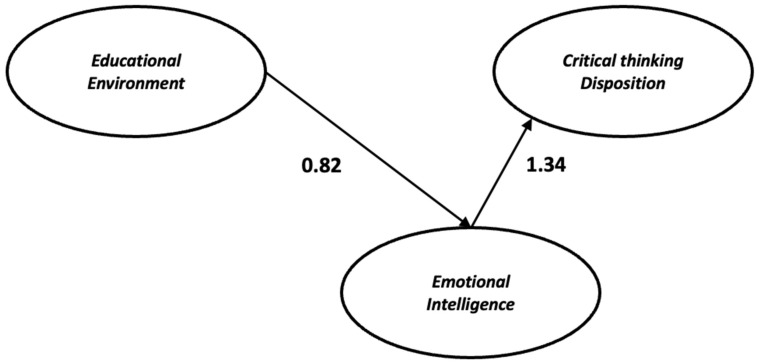
The effect of learning environment and emotional intelligence on critical thinking. Numbers next to the arrows are beta coefficients (β).

Finally, the learning environment had a positive indirect effect (β = 1.34, *p* = 0.024) on critical thinking disposition (Table 3) through emotional intelligence (Figure 2). More specifically, bootstrapping results (5000 bootstrap samples with 95% confidence intervals) demonstrated that the indirect effect does not contain zero (0.132–2.17) (Table 3). 

## 4. Discussion

In the present study, we examined the indirect relationship between the learning environment and critical thinking through students’ emotional intelligence. The results demonstrate that the learning environment was positively related to emotional intelligence and the latter was associated with critical thinking disposition (Table 3). Furthermore, we found that the learning environment only has an indirect relationship with critical thinking via emotional intelligence (Figure 2). 

To our knowledge, this is the first study that examines the mediating role of emotional intelligence in the relationship between the learning environment and critical thinking. This finding has two major implications: one for healthcare educators and another for researchers. First, educators could utilize learning methods that increase emotional intelligence and consequently improve critical thinking disposition. Second, researchers could explore other potential modifiable factors of critical thinking and their possible relationships with the learning environment.

Our study established a positive relation between emotional intelligence and critical thinking (Table 3). This coincided with the findings of another cross-sectional study on 500 first year students that used two different questionnaires to measure emotional competence and critical thinking [23]. Another cross-sectional study of 296 participants showed that people with a higher emotional intelligence used emotional information more efficiently, therefore improving critical thinking [22]. Additionally, a systematic review concluded that critical thinking was correlated to emotional intelligence, a finding similar to ours [60]. Furthermore, a cross sectional study of 269 medical students suggested a positive correlation between emotional intelligence, critical thinking, and the management skills of medical students [61]. A possible explanation for these findings could be that emotions influence human behavior, and, since critical thinking disposition is a behavior, It could be affected by emotional intelligence [23]. However, a longitudinal study of 197 nursing students, which measured critical thinking and emotional intelligence with different questionnaires to our study, found that critical thinking and emotional intelligence were not associated, and that they did not improve over an academic year [62]. Additionally, a quantitative, descriptive–correlative study of 169 nursing students, which also used different questionnaires to our study, concluded that critical thinking could be affected only by one component of emotional intelligence, which was empathy [21]. These differences with our findings could be attributed to the differences in the sample and methodology. In addition, regarding the validity and reliability of the questionnaires, Cronbach’s a values for the overall scales that were used in this study were well above the recommended 0.70 threshold, with the exception of the reflective skepticism of the CTDS subscale (a = 0.60). Although it does not affect our results, four additional subscales (social of DREEM, and self-control, emotionality, and sociability of TEIQue-SF) were in the range of 0.57–0.70. According to Tavakol and Dennick [58], Cronbach’s a values are affected by the correlation and the number of items in a test, as well as the sample of those tested. This could explain the lower Cronbach’s a values in these subscales. In general, all three questionnaires used in this study have shown comparable internal reliability in other studies [46,63,64].

Contrary to our first hypothesis, we found that the learning environment was not directly associated with critical thinking (Table 3). However, other studies have shown a positive relationship between the learning environment and critical thinking. For example, a qualitative analysis of interviews of 44 expert teachers implied that the learning environment proved to be beneficial in developing the critical thinking of each student [65]. Additionally, a qualitative study on 13 faculty teachers and 44 dental students observed that, when the learning environment included clinical exercises, which were carefully planned, critical thinking could be promoted [66]. Finally, a quasi-experimental study on 170 nursing students, which evaluated critical thinking before and after a two-year course intervention, suggested that the learning environment could increase critical thinking [67]. This variation in our results could be attributed to the sample (first year students) of the study, who might need more time to cultivate their critical thinking in a new learning environment. However, the choice of first years was deliberate because the sooner educators identify any shortcomings, the better, since they can overcome them more quickly. Furthermore, we chose the first years so as to longitudinally depict and intervene on any shortcomings throughout their educational years. 

To our knowledge, this is the first collaborative study in Greece between three universities (two nursing departments and one medicine department) to explore and then improve the critical thinking of their students. A strong point of our study was that emotional intelligence was identified as a strong mediator between the learning environment and critical thinking. Consequently, educators could potentially utilize this knowledge and apply methods to improve students’ emotional intelligence, thus also improving their critical thinking, and later optimizing the quality of care that they provide.

## 5. Limitations

The present study has a few limitations. First, ascertaining causal relationships is difficult due to the one-time measurement, although this is common in cross-sectional studies [68] and the directions of the associations that we propose are the most plausible. Longitudinal studies are needed to establish possible causal relationships. Additionally, common method variance due to the data collection process cannot be excluded [69]. Another limitation was that the questionnaire that we used to assess EI measured it as a trait and not as a performance-based skill. Additionally, the sample of the study consisted of only first-year health sciences students (mostly nursing) from three Greek universities, the majority of which were female (74.5%), thus limiting the generalizability of our findings. It should be noted, however, that, especially for the nursing students, our study population corresponds to the general nursing student population in Greece. Future studies could also examine and compare the present hypotheses between the two groups of nursing and medicine students and highlight potential differences. Finally, emotional intelligence was measured as a personality trait that some authors claim is genetically predetermined, and therefore may not be affected by the learning environment [70]. However, a meta-analysis showed that the “affective perspective taking” component of emotional intelligence can be cultivated through the learning environment [70].

## 6. Conclusions

In conclusion, we provided compelling evidence that the learning environment has an indirect positive effect on critical thinking disposition through emotional intelligence. Therefore, healthcare educators could utilize methods that cultivate the emotional intelligence of their students to improve their critical thinking disposition. This evidence also suggests that universities should adapt their curricula and further improve the quality of their learning environments, thus increasing the emotional intelligence of their students. This would further increase critical thinking and lead to a higher quality of care by future healthcare professionals. 

## Figures and Tables

**Table 1:** Demographic characteristics of the 208 participants.

		n	%
**Gender**	Male	53	25.5
	Female	155	**74.5**
**Age, years**	18	71	34.1
	19	84	40.4
	20	24	11.6
	21+	29	13.9
	mean ± stand. dev. (min, max)	20.8 ± 6.5 (18.0, 54.0)	
**University School**	Medicine	65	31.3
	Nursing	143	**68.7**

**Table 2:** Score levels of Critical Thinking Disposition Scale (CTDS), Dundee Ready Education Environment Measure (DREEM), and Trait Emotional Intelligence Questionnaire Short Form (TEIQue-SF).

Scales and Subscales	Mean Score	Stand. Dev.	Median	Min, Max	Cronbach’s α
**Critical Thinking Disposition Scale *(CTDS)***	**44.5**	4.9	44.0	17, 66	**0.78**
**Critical Openness (seven items)**	29.3	3.5	29.0	9, 42	0.753
**Reflective Skepticism (four items)**	15.2	2.2	15.0	8, 24	0.601
**Dundee Ready Education Environment Measure *(DREEM)***	**124.4**	20.4	123.0	64, 183	***0.933***
**Learning**	29.0	5.9	29.0	10, 48	0.853
**Teachers**	26.5	4.8	26.0	13, 41	0.763
**Academic**	21.3	4.0	21.0	8, 31	0.729
**Atmosphere**	29.8	6.0	30.0	10, 46	0.806
**Social**	17.8	3.3	18.0	6, 26	0.597
**Trait Emotional Intelligence Questionnaire Short Form (TEIQue-SF)**	**4.92**	0.66	4.96	2.53, 6.63	**0.823**
**Well-being**	5.25	1.09	5.50	2.00, 7.00	0.795
**Self-control**	4.40	1.03	4.50	1.00, 6.83	0.638
**Emotionality**	5.03	0.82	5.13	2.88, 6.75	*0.570*
**Sociability**	4.66	0.95	4.67	1.00, 7.00	0.632

**Table 3:** Regression analyses between Critical Thinking Disposition Scale (CTDS), Dundee Ready Education Environment Measure (DREEM), and Trait Emotional Intelligence Questionnaire Short Form (TEIQue-SF).

	Emotional Intelligence Regressed on Learning Environment	Critical Thinking Disposition Regressed on Emotional Intelligence, Controlling for Learning Environment	Critical Thinking Disposition Regressed on Learning Environment, Controlling for Emotional Intelligence	Bootstrap Results for Indirect Effect	
**Critical thinking disposition—Overall**					
β	0.82	**1.34**	1.06	M	**1.10**
SE	0.11	0.59	1.01	SE	0.52
t	7.74	2.28	1.05	L95% CI	**0.13**
*p*	<0.001	**0.024**	*0.30*	U95% CI	**2.17**
**Critical thinking disposition—Critical Openness**					
β		0.68	1.07	M	0.56
SE		0.42	0.72	SE	0.33
t		1.63	1.49	L95% CI	−0.059
*p*		0.10	0.14	U95% CI	1.25
**Critical thinking disposition—Reflective Skepticism**					
β		0.66	−0.01	M	0.54
SE		0.27	0.46	SE	0.26
t		2.44	−0.02	L95% CI	0.05
*p*		0.02	0.99	U95% CI	1.08

Note. Unstandardized regression coefficients reported. Bootstrap sample size 5000. β = Beta coefficients; SE = standard error; L = lower limit; U = upper limit; CI = confidence interval.

## Data Availability

The data presented in this study are available on request from the corresponding author. The data are not publicly available due to privacy restrictions.
